# Acupuncture Modulates Disrupted Whole-Brain Network after Ischemic Stroke: Evidence Based on Graph Theory Analysis

**DOI:** 10.1155/2020/8838498

**Published:** 2020-08-19

**Authors:** Xiao Han, He Jin, Kuangshi Li, Yanzhe Ning, Lan Jiang, Pei Chen, Hongwei Liu, Yong Zhang, Hua Zhang, Zhongjian Tan, Fangyuan Cui, Yi Ren, Lijun Bai, Yihuai Zou

**Affiliations:** ^1^Department of Neurology and Stroke Center, Dongzhimen Hospital, The First Affiliated Hospital of Beijing University of Chinese Medicine, Beijing, China; ^2^The National Clinical Research Center for Mental Disorders & Beijing Key Laboratory of Mental Disorders, Beijing Anding Hospital, Capital Medical University, Beijing, China; ^3^Department of Neurology, Shunyi Hospital Affiliated to Beijing Hospital of Traditional Chinese Medicine, Beijing, China; ^4^Department of Radiology, Dongzhimen Hospital, The First Affiliated Hospital of Beijing University of Chinese Medicine, Beijing, China; ^5^The Key Laboratory of Biomedical Information Engineering, Ministry of Education, Department of Biomedical Engineering, School of Life Science and Technology, Xi'an Jiaotong University, Xi'an, China

## Abstract

**Background:**

Stroke can lead to disruption of the whole-brain network in patients. Acupuncture can modulate the functional network on a large-scale level in healthy individuals. However, whether and how acupuncture can make a potential impact on the disrupted whole-brain network after ischemic stroke remains elusive.

**Methods:**

26 stroke patients with a right hemispheric subcortical infarct were recruited. We gathered the functional magnetic resonance imaging (fMRI) from patients with stroke and healthy controls in the resting state and after acupuncture intervention, to investigate the instant alterations of the large-scale functional networks. The graph theory analysis was applied using the GRETNA and SPM12 software to construct the whole-brain network and yield the small-world parameters and network efficiency.

**Results:**

Compared with the healthy subjects, the stroke patients had a decreased normalized small-worldness (*σ*), global efficiency (*E*_g_), and the mean local efficiency (*E*_loc_) of the whole-brain network in the resting state. There was a correlation between the duration after stroke onset and *E*_loc_. Acupuncture improved the patients' clustering coefficient (*C*_p_) and *E*_loc_ but did not make a significant impact on the *σ* and *E*_g_. The postacupuncture variables of the whole-brain network had no association with the time of onset.

**Conclusion:**

The poststroke whole-brain network tended to a random network with reduced network efficiency. Acupuncture was able to modulate the disrupted patterns of the whole-brain network following the subcortical ischemic stroke. Our findings shed light on the potential mechanisms of the functional reorganization on poststroke brain networks involving acupuncture intervention from a large-scale perspective.

## 1. Introduction

Following a stroke, focal ischemic lesions can result in extensive functional changes in structurally intact brain areas far beyond the infarct, which is referred to as “diaschisis” [1–3]. The cataclysmic changes in the whole-brain network are attributable to the widespread disturbance following a focal injured site [4, 5]. These large-scale interactions between brain regions account for the behavioral deficits in patients with stroke [6]. The normalization of an abnormal connectome has been found to mirror the recovery from stroke [7, 8]. Graph theory-based network analysis is widely used to characterize the features of the large-scale brain connectome [9–11]. The small-world model is well suited for the evaluation of complex brain networks since it reflects the integrative and segregated information processes [12, 13]. The focal lesions may disturb the spatial and temporal organization and break the optimal balance of integration and segregation [14].

Based on graph theoretical approaches, previous studies emphasized that the small-worldness was significantly lower in stroke patients than healthy controls, and it increased toward the level of controls during recovery [8]. The reductions in interhemispheric integration and intrahemispheric segregation strongly relate to the behavioral impairments of stroke [15]. In subcortical stroke patients, the reorganized network deviated away from the optimal architecture toward a more random mode during the first months after the stroke onset [16]. However, the reorganization of the entire brain pattern during stroke recovery is still disputable and elusive for now.

Acupuncture is a well-known therapeutic strategy in China for over two millennia of practice, which is also widely accepted in modern Western countries [17, 18]. Increasingly, empirical and clinical evidence suggests that acupuncture is effective and safe for subacute stroke rehabilitation [19, 20]. According to the theory of Chinese medicine, acupuncture can dynamically harmonize the inherent imbalances that result from diseases. Besides, acupuncture is more inclined to modulate the homeostasis of brain activity at a large scale [21].

The previous study identified that acupuncture altered the architecture of the functional whole-brain network in healthy subjects and the alterations displayed acupoint specificity [22–24]. Acupuncture stimulation at acupoint ST36 increased the local efficiency of healthy individuals [25]. Furthermore, another study applied the long-duration transcutaneous electric acupoint stimulation and indicated that small-world properties were modulated with lower local efficiency and nonsignificant change in global efficiency for healthy subjects [26]. But the therapeutic effects of acupuncture may depend on different conditions of clinical diseases [23, 25]. Feng et al. studied the acupuncture effects on the functional network of the whole brain in mild cognitive impairment patients [27]. They found that acupuncture had a relatively less effect on healthy controls than patients. With the help of graph theory analysis, it revealed some previously unreported features of the neural mechanism after acupuncture on patients.

The architecture of brain after subcortical infarction remains poorly understood. Moreover, little is known about the alternations in response to acupuncture on the large-scale perspective after stroke onset. The current study, therefore, employed the graph theoretical approaches to investigate how acupuncture affected the whole-brain functional network following a subcortical insult. We hypothesized that acupuncture was able to modulate the deviant organization of the poststroke whole-brain network.

## 2. Materials and Methods

### 2.1. Subjects

26 ischemic stroke patients were recruited at Dongzhimen Hospital affiliated to Beijing University of Chinese Medicine. 21 healthy subjects were rolled as control with no history of neurological or psychiatric disorders. The demographic and clinical features are shown in Table 1. The inclusion criteria were as follows: aged 35-75 years; first-ever ischemic stroke; within 3 months after the onset; subcortical lesion restricted to the right hemispheric internal capsule, basal ganglia, corona radiate, and its neighboring regions; and without psychiatric disorders. The exclusion criteria were as follows: any brain abnormalities except infarction; any other physical or psychiatric conditions that may influence participation; and any MRI contraindications. All of the subjects were with right-hand dominance. The study was approved by the Ethics Committee of the Beijing University of Chinese Medicine and conducted in accordance with the Declaration of Helsinki. After being given a complete explanation of the experiment procedure, all subjects signed the informed consent.

### 2.2. Clinical Assessments

The National Institute of Health Stroke Scale (NIHSS) was performed for stroke-related neurologic deficits [28] and the Fugl-Meyer Assessment (FMA) was adopted for a quantitative measure of motor disability [29]. All patients underwent these clinical assessments (shown in Table 1).

### 2.3. Experimental Paradigm

The nonrepeated event-related fMRI design was adopted to investigate the prolonged effects of acupuncture administration [30]. Firstly, a resting-state scan was conducted for 8 min 10 sec without any stimulation as a baseline control. Then, after a DTI session, we employed an experimental functional run under acupuncture stimulation. The acupuncture stimulation was delivered using a sterile disposable silver acupuncture needle 0.3 mm in diameter and 40 mm in length. The needle was vertically inserted to a depth of 20-30 mm at the acupoint GB34 (Yanglingquan) on the right side. The GB34 situates on the fibular aspect of the leg, in the depression anterior and distal to the head of the fibula. It is commonly used in the clinical treatment for stroke [19, 31, 32]. The acupuncture administration was delivered by a balanced “tonifying and reducing” manipulation (rotating the needle clockwise and counterclockwise at 1 Hz for 60 sec) and followed by an 8 min 10 sec consecutive scan without manipulation. The procedure was performed by the same licensed and experienced acupuncturist. All subjects reported their experience (“Deqi”) of acupuncture stimulation immediately after the experiment. “Deqi” included the sensations of soreness, heaviness, fullness, pressure, and numbness [33]. The subject who experienced sharp pain would be excluded from further analysis as the sharp pain was considered an inadvertent noxious stimulation [34]. None of the subjects reported an experience of sharp pain. In this study, none of the subjects ever received thrombolytic therapy. All of the patients received conventional standard medical treatment, such as antiplatelet therapy, which complied with the *Guidelines for the diagnosis and treatment of acute ischemic stroke in China 2014* [35].

### 2.4. Image Acquisitions

The fMRI data were obtained using a 3.0 Tesla MRI scanner (Siemens, Sonata Germany) at the department of radiology of Dongzhimen Hospital, Beijing, China. During the scanning, all subjects were requested to keep their eyes closed and remain relaxed without engaging in any mental tasks. Earplugs were worn to attenuate scanner noise, and foam head holders were immobilized to minimize head movements.

Before the functional scanning, high-resolution structural information for anatomical localization was acquired using 3D MRI sequences. A single-shot, gradient-recalled echo-planar imaging sequence was used to collect the resting-state fMRI data with the following parameters: repetition time = 2000 ms, echo time = 30 ms, flip angle = 90°, matrix = 64 × 64, field of view = 225 × 225 mm^2^, slice thickness = 3.5 mm, gap = 1 mm, 32 interleaved axial slices, and 241 volumes. The same parameters were applied in the acupuncture-evoked fMRI with the exception that 271 volumes were acquired.

### 2.5. Data Preprocessing

The fMRI data were preprocessed with the Graph Theoretical Network Analysis (GRETNA) (http://www.nitrc.org/projects/gretna) and SPM12 (http://www.fil.ion.ucl.ac.uk/spm) toolbox based on Matlab2013b [36]. The first ten volumes were discarded to allow the adaption of the subjects and the stabilization of the magnetization. The remaining volumes were slice-timing corrected for different acquisition in slice times and realigned to the first volume for head-motion correction. Based on the head motion data, the subjects were excluded according to the criteria of maximum translation as 3 mm and rotational parameters as 3 degrees in any direction. Next, the individual functional images were normalized to the standard Montreal Neurological Institute space with 3 mm isotropic resolution by applying the transformation matrix which was derived from registering the final template file generated by DARTEL. Then, the images were smoothed with a 4 mm full-width at the half-maximum Gaussian kernel and further linearly detrended. Subsequently, several nuisance signals were regressed out, including the white matter signal, the cerebrospinal fluid signal, and 24-parameter head motion profiles [37, 38]. The global signal was not regressed out according to previous studies [39]. Then, the data were temporally band-pass filtered (0.01-0.08 Hz) to reduce the low-frequency drift and high-frequency physiological noise.

### 2.6. Network Construction

The graph theoretical network analysis toolbox GRETNA was applied to construct the large-scale brain networks [36]. For the network nodes definition, the entire cerebral cortex was parceled into 90 (45 in each hemisphere) anatomically defined regions according to Automated Anatomical Labeling for each subject [40]. The mean time series for each of the 90 areas were extracted from the preprocessed datasets by averaging the voxel time series within each region. The edges of functional brain networks were constructed with the functional connectivity between nodes. Pearson's correlation coefficients between the mean time series of all node pairs were calculated, resulting in a 90 × 90 correlation matrix for each subject. Fisher's *r*-to-*z* transformation was further performed to improve the normality, and this resulted in a *z*-value matrix for each subject. The matrices were binary, and both positive and negative connections were used to achieve the whole-brain network.

### 2.7. Networks Analysis

We employed a wide range of sparsity thresholds (0.05 ≤ sparsity ≤ 0.5, interval = 0.01) to address a variable number of edges in different individual subjects. The sparsity is defined as the existing number of edges divided by the maximum possible number of edges in the graph [41, 42]. The global metrics contained the small-world parameters and network efficiency to depict the entire brain functional connectomes. The main small-world parameters included the clustering coefficient (*C*_p_), characteristic path length (*L*_p_), and small-worldness (*σ*). The network efficiency included global efficiency (*E*_g_) and the mean local efficiency (*E*_loc_). The mean local efficiency was defined as the average efficiency of the local subgraphs. A detailed explanation of these network parameters can be found in the previous study [43]. The area under the curve (AUC) was also calculated for each network metric to provide a summarized scalar. The AUC was independent of a single threshold selection and has been proved to be highly sensitive to topological alterations in brain disorders [44, 45].

### 2.8. Statistical Analysis

The demographics and clinical profiles were analyzed by two-sample *t*-test for continuous variables and Chi-square test for categorical variables using the SPSS software version 19.0 (http://www.spss.com; Chicago, IL). The statistical comparisons of measures (*C*_p_, *L*_p_, *σ*, *E*_g_, and *E*_loc_) and their AUC were performed using independent two-sample *t*-test between stroke patients and healthy controls with age and gender as unconcerned covariates. We applied the paired *t*-test to determine the pre- and proacupuncture alterations of these large-scale network matrices. We also performed a two-tailed Spearman's rank correlation between the AUC of parameters and the clinical measures in the stroke patients. *p* < 0.05 was considered to be statistically significant.

## 3. Result

### 3.1. Demographic and Clinical Characteristics

In the present study, the subcortical ischemic lesions were restricted to the motor pathways of the right hemisphere. The time of stroke onset was 41.04 ± 29.71 days. There were no statistical differences in age (*p* = 0.315) or sex (*p* = 0.355) between the patients and healthy controls. The average score of National Institute of Health Stroke Scale (NHISS) was 3.46 ± 2.58, and Fugl-Meyer Assessment (FMA) was 78.20 ± 23.46. Table 1 summarizes the demographic and clinical characteristics of all the subjects.

### 3.2. Small-World Parameters at Resting State

**Figure 1:**
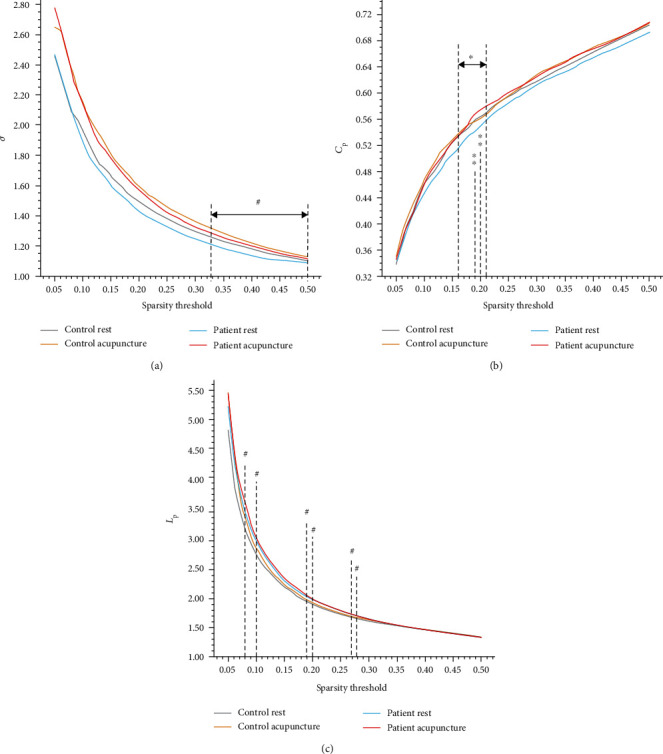
Comparison of small-world parameters (*σ*, *C*_p_, and *L*_p_) at a sparsity range of 0.05-0.50. (a) The small-worldness (*σ*) was compared between the stroke patients and the healthy controls, as well as the resting-state and postacupuncture intervention. In the resting state, the patients with stroke exhibited significantly lower *σ* values relative to the healthy controls on a sparsity threshold from 0.33 to 0.50. However, after acupuncture intervention, the enhanced *σ* values were not statistically different from the resting state. (b) On the sparsity threshold of 0.16-0.21, the clustering coefficient of patients was notably improved by acupuncture. (c) At several separated sparsities (Sp = 0.08, 0.10, 0.19, 0.20, 0.27, 0.28), there were statistical differences in the characteristic path length between the two groups of subjects in the resting state. ^#^*p* < 0.05 (stroke patients vs. healthy controls, in the resting state); ∗*p* < 0.05, ∗∗*p* < 0.01 (resting state vs. postacupuncture intervention, in the stroke patients).

**Figure 2:**
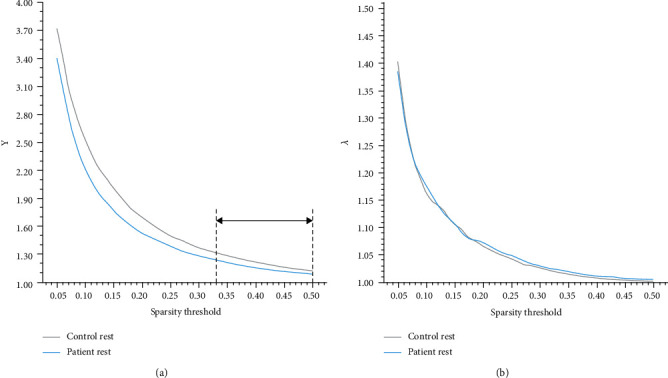
Comparison of the normalized small-world parameters (*γ*, *λ*). (a) The values of normalized clustering coefficient (*γ*) decreased in the stroke patients relative to healthy controls on the sparsity threshold of 0.33-0.50. (b) There was no significant difference in the normalized characteristic path length (*λ*) between two groups of subjects. ^#^*p* < 0.05.

**Figure 3:**
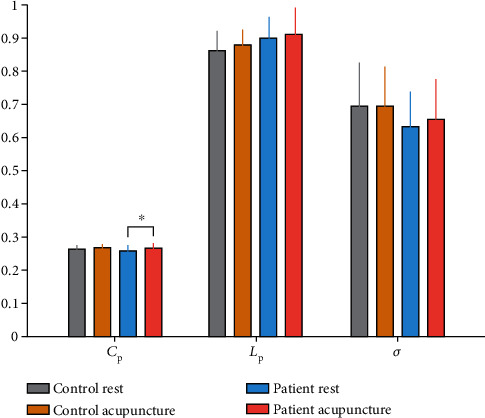
Mean area under the curve (AUC) values of the small-world parameters. The stroke patients displayed no marked discrepancy in contrast with the healthy controls on the AUC. Acupuncture intervention made a distinct improvement on the AUC of *C*_p_ values in the stroke patients. ∗*p* < 0.05.

The matrices were constructed with a wide range of sparsity (0.05 ≤ Sp ≤ 0.5, in 0.01 increments) in all subjects. We calculated the small-world parameters and compared them between the stroke and healthy subjects. Although both groups met the criteria of the small-worldness (*σ* > 1), the *σ* of the stroke patients had a significant reduction relative to healthy subjects (0.33 ≤ Sp ≤ 0.50, shown in Figure 1(a)). At the same time, the values of *L*_p_ increased at several separated sparsity in stroke 4individuals (shown in Figure 1(c)), but the *C*_p_ did not have statistic discrepancy (shown in Figure 1(b)). To further investigate the potential causes of the decline in *σ*, we analyzed the normalized clustering coefficient (*γ* = *C*_p_/*C*_p_ random) and the normalized characteristic path length (*λ* = *L*_p_/*L*_p_ random), by calculating which would get the small-worldness (*σ* = *γ*/*λ*). The stroke patients displayed a distinct decrease on the parameter *γ* in contrast with the healthy controls (0.33 ≤ Sp ≤ 0.50, shown in Figure 2(a)), while they did not show the difference on the *λ* (shown in Figure 2(b)). The AUC of the small-world parameters had no significant difference between stroke patients and healthy controls (shown in Figure 3).

### 3.3. Acupuncture Altered Small-World Parameters

We analyzed the alterations of matrices before and after acupuncture intervention in patients with stroke and the healthy controls. The *σ* value did not show a statistical improvement after needling. However, there was a trend of increased *σ* in postacupuncture stroke patients (shown in Figure 1(a)). The acupuncture significantly increased the *C*_p_ on the sparsity threshold of 0.16-0.21 (shown in Figure 1(b)). Also, the AUC of *C*_p_ was also conspicuously increased after acupuncture intervention relative to the resting state in the stroke patients (*p* = 0.041, shown in Figure 3). There were no significant changes in the parameter *L*_p_ after acupuncture intervention (shown in Figure 1(c)).

### 3.4. Network Efficiency

**Figure 4:**
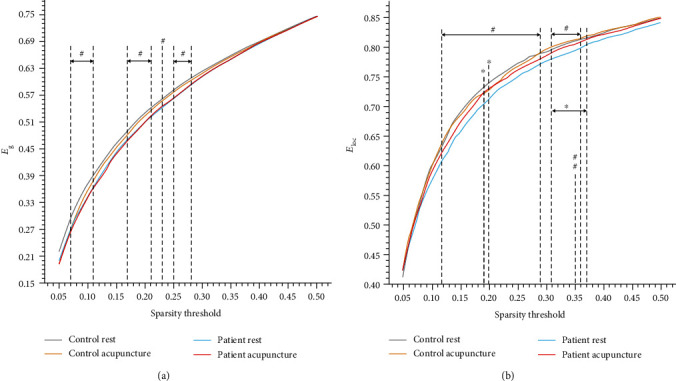
Comparison of the global (*E*_g_) and local (*E*_loc_) efficiency at a sparsity range of 0.05-0.50. (a) The stroke patients showed a decline in the global efficiency relative to the healthy controls at certain sparsity (Sp = 0.07 − 0.11, 0.17 − 0.21, 0.23, 0.25 − 0.28). (b) The patients exhibited a conspicuous damage on the local efficiency than the healthy controls (Sp = 0.12 − 0.29, 0.31 − 0.36), while acupuncture intervention showed distinct alteration on the local efficiency of the stroke patients (Sp = 0.19, 0.20, 0.31 − 0.37). ^#^*p* < 0.05, ^##^*p* < 0.01 (stroke patients vs. healthy controls, in the resting state); ∗*p* < 0.05 (resting state vs. postacupuncture intervention, in the stroke patients).

**Figure 5:**
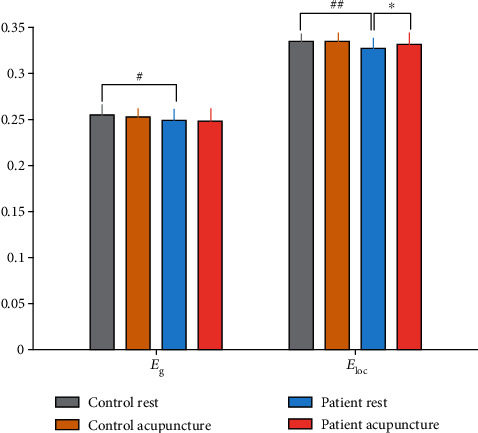
Mean area under the curve (AUC) values of the network efficiency. The patients had a significant reduction on the AUC value of both the global and local efficiency in the resting state compared with the healthy controls. Acupuncture intervention was able to make a noteworthy increase on the local efficiency, while having no distinct impact on the global efficiency. ∗*p* < 0.05 (stroke patients vs. healthy controls, resting state). ^#^*p* < 0.05, ^##^*p* < 0.01 (stroke patients vs. healthy controls, in the resting state); ∗*p* < 0.05 (resting state vs. postacupuncture intervention, in the stroke patients).

The between-group comparison revealed that the stroke patients exhibited notably lower *E*_g_ and *E*_loc_ than the healthy controls on certain sparsity thresholds (shown in Figures 4(a) and 4(b)). The AUC of network efficiency also testified a significant diminishment on the *E*_g_ (*p* = 0.045) and *E*_loc_ (*p* = 0.008) in the stroke patients (shown in Figure 5). The acupuncture intervention had no noticeable impact on the *E*_g_, but markedly enhanced the *E*_loc_ at certain sparsity (shown in Figure 4) and the AUC of *E*_loc_ (*p* = 0.032, shown in Figure 5) in the patients with stroke.

### 3.5. Correlations between Graph Theory Metrics and Clinical Measures

**Figure 6:**
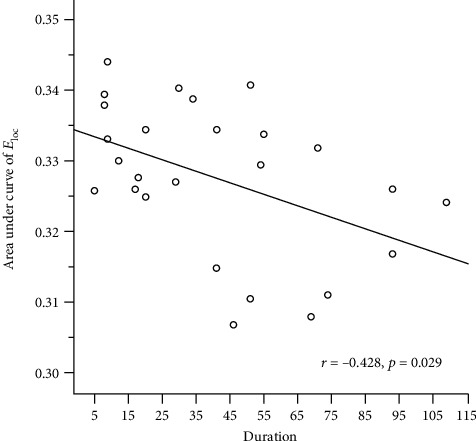
The Spearman rank correlation between the AUC of local efficiency and time of stroke onset. There was a significant negative association between the AUC of *E*_loc_ and the duration after stroke onset (*p* = 0.029).

We further analyzed the associations between the AUC of graph theory metrics and the clinical scores in the stroke patients. A significant negative correlation between the AUC of *E*_loc_ and the time of stroke onset was found (*p* = 0.029, shown in Figure 6

## 4. Discussion

In this study, we applied the graph-based theoretical approaches to analyze the properties of the entire brain. The functional whole-brain network of the stroke patients and healthy controls before and after acupuncture intervention were constructed separately. Our results revealed that the large-scale functional patterns in both groups of subjects exhibited the small-world features; however, the patients with stroke displayed a declined on the normalized clustering coefficient (*γ*), small-worldness (*σ*), global efficiency (*E*_g_), and the mean local efficiency (*E*_loc_) at resting state in contrast with the healthy individuals. The reduced local efficiency was correlated with the duration after stroke onset. Acupuncture significantly improved the clustering coefficient and the mean local efficiency of the stroke individuals but did not make a statistic impact on the small-worldness or global efficiency. This study primarily demonstrated the modulation of acupuncture on the poststroke functional whole-brain network.

In the current study, the small-worldness was conspicuously declined in the stroke patients contrasted to the healthy subjects. To further study it, we analyzed the parameters *γ* and *λ*. In general, the usual small-world networks have relatively high *γ* and low *λ*, which combine the topological properties of both random and regular networks to maintain simultaneously high ability in segregating and integrating information. The integration and segregation are two major principles of the human brain functional organization. We found that the parameter *γ* was notably decreased in the stroke patients in the same range of sparsity, which implied that the *γ* was supposed to be responsible for the decline of the small-worldness. The *γ* was the normalized version of clustering coefficient and reflected the ability in segregating information. These changes of the large-scale patterns result from ischemic infarction which were in line with a previous study [8]. Siegel et al. showed that the small-worldness decreased after stroke onset, and they also inferred that the poststroke changes of small-worldness were a result of changes in the clustering coefficient, but not the average path length. Our findings indicated that the stroke attack altered the architecture of the functional whole-brain network. The breakdown of human brain function in stroke might be mainly involved in the segregation instead of the integration, which shifted the poststroke whole-brain network toward a random graph. Zhu et al. also reported similar results. They indicated a tendency of randomization in acute ischemic stroke patients' functional brain networks [46].

The network efficiency is an assessment of how efficiently a network exchanges information [47]. At the global level, efficiency quantifies the effectiveness of integration of distributed information across the whole-brain network where information is concurrently exchanged [48]. It reflects the capacity for network-wide communication [41]. The local efficiency relies on the connectivity properties in the neighborhood of a node and indicates how efficient is the communication between a region and its neighboring areas [49]. The local efficiency is the metric that reflects the fault tolerant [47]. In the present study, not only the global efficiency but also the mean local efficiency was significantly lower in the stroke patients relative to the healthy subjects, which were in agreement with the findings of small-worldness alterations. These results revealed that the focal ischemic insult not only disrupted the ability to exchange information through the entire network but also minified the overall level of the communication within the neighborhoods. An optimal brain achieves a dynamic balance between global integration and local specialization with both relatively high global and local efficiency [50]. Some previous findings also unveiled that the brain regions of the poststroke networks communicated less efficiently both on the global and local range [15].

Our study for the first time manifested that acupuncture was able to make an instant improvement on the poststroke clustering coefficient (*C*_p_), and the reduced local efficiency was also escalated. However, neither the small-worldness nor the global efficiency had significant modifications under the acupuncture intervention. It hinted that acupuncture enhanced the connection within the neighborhood regions and modulated the local information flow, which could participate in the reorganization of the poststroke brain network and the process of neural plasticity. We supposed that the acupuncture might mainly work on the recovery of the segregation function in the whole-brain network after a stroke attack. Acupuncture might be able to promote the organization of poststroke network toward a regular one. In addition, the above-mentioned alterations were not observed within the healthy subjects. It implied that acupuncture could mediate the poststroke whole-brain networks, and this specific modulation arose under the condition of stroke. Nevertheless, in the present study, we focused on the instant effect of acupuncture instead of the sustained effect after a period of acupuncture treatments and just intervened on a single acupoint instead of a group of acupoints. This strategy might result in that the acupuncture intervention was not enormous enough to make statistically significant alterations on the small-worldness or the global efficiency. It is still not sure whether acupuncture can also make an influence on the small-worldness and global efficiency if we adopted a longitudinal acupuncture intervention with multiple acupoints involved. In the future, we would progress to the long-term acupuncture efficacy with various acupoints combination. It might be able to uncover more impact of acupuncture treatment on the whole-brain network. In addition, this study focused on the functional alterations on the large scale. Further investigation could combine the function and structure data of the whole-brain network to have a better understanding of the strategy of the brain responding to stroke and acupuncture.

The correlations analysis showed a significant negative association between the duration after stroke onset and the AUC of the mean local efficiency. We speculated that for the stroke patients within 3 months of onset, the local efficiency might gradually decrease over time, which might be due to the pathological dynamics after a stroke attack, such as the Wallerian degeneration. It might indicate that the application of acupuncture in the early stage of stroke can make more benefits for stroke patients, which needs to be further confirmed. The NIHSS and FMA scores showed no correlation with the AUC of the small-worldness or the network efficiency. Additionally, the parameters of the postacupuncture network matrices in the stroke patients did not associate with the clinical measures in our study. However, as this study included ischemic stroke patients within 3 months after the stroke onset, the dynamic patterns in a longer term still need further study. In future research, the stroke phase, combining the time of onset and the degree of the neurological deficits, can be taken as an important factor to uncover the different characteristics within stroke patients.

## 5. Conclusion

The present work pointed to the functional disruption of the whole-brain network in the stroke patients and the acupuncture modulation. In short, we illustrated that the stroke attack was able to reduce the small-worldness and the global and the mean local efficiency; at the same time, acupuncture can instantly increase the clustering coefficient and the mean local efficiency of the whole-brain network but not make statistically significant alterations on the small-worldness or the global efficiency. This study highlighted that acupuncture could help reorganize the disrupted poststroke whole-brain network. Our study provided an elucidation for the mechanisms of acupuncture treatment after stroke from the large-scale perspective.

## Figures and Tables

**Table 1:** The group demographic and relevant clinical measures.

Subject	Sex	Age (yrs.)	Time of onset (*d*)	NHISS	FMA
Patient	19M/7F	56.42 ± 7.23	41.04 ± 29.71	3.46 ± 2.58	78.20 ± 23.46
Control	12M/9F	54.52 ± 5.11	n/a	n/a	n/a

F: female; FMA: Fugl-Meyer Assessment; M: male; NIHSS: National Institute of Health Stroke Scale.

## Data Availability

The datasets generated and analyzed during the current study are not publicly available due to the terms of consent to which the participants agreed but are available from the corresponding author on reasonable request.
